# Association between different GLP-1 receptor agonists and acute pancreatitis: case series and real-world pharmacovigilance analysis

**DOI:** 10.3389/fphar.2024.1461398

**Published:** 2024-11-13

**Authors:** Hui Guo, Qian Guo, Zhiqiang Li, Ze Wang

**Affiliations:** ^1^ Department of Pharmacy, Shanxi Cardiovascular Disease Hospital, Taiyuan, Shanxi, China; ^2^ Department of Pharmacy, Second Hospital of Shanxi Medical University, Taiyuan, Shanxi, China; ^3^ Department of Pharmacy, Shanxi Traditional Chinese Medical Hospital, Taiyuan, Shanxi, China

**Keywords:** GLP-1 receptor agonists, acute pancreatitis, FAERS, pharmacovigilance, data mining

## Abstract

**Objective:**

Glucagon-like peptide-1 receptor agonists (GLP-1 RAs) have shown notable advancements in managing blood sugar control. Nevertheless, there remains a gap in real-world data regarding the variation in acute pancreatitis (AP) risk among different GLP-1 RAs. Our study aimed to characterize and evaluate AP associated with different GLP-1 RAs (exenatide, lixisenatide, liraglutide, albiglutide, semaglutide, dulaglutide and tirzepatide) in a public adverse events database and to review the relevant case reports.

**Methods:**

We described a case series of patients experiencing AP while on GLP-1 RAs. Additionally, we utilized various algorithms including reporting odds ratio (ROR), proportional reporting ratio (PRR), Bayesian confidence propagation neural network (BCPNN), and multi-item gamma Poisson shrinker (MGPS) to analyze data from the Food and Drug Administration’s Adverse Event Reporting System (FAERS) regarding suspected adverse events of AP linked to GLP-1 RAs from January 2005 to September 2023.

**Results:**

Our case series comprised thirty-nine patients who experienced AP events while on GLP-1 RAs. Within the FAERS database, we retrieved a total of 6,751 individual case safety reports (ICSRs) involving various GLP-1 RAs. The median age of the patients included in our study was 57 years (range: 14–99), with 98.3% of cases classified as serious. Signals indicating AP were observed across all GLP-1 RAs, with particular emphasis on exenatide and liraglutide.

**Conclusion:**

There is a notable reporting signal of AP associated with all GLP-1 RAs. Healthcare providers must remain vigilant and closely monitor this potentially life-threatening adverse event.

## 1 Introduction

Globally, over 95% of diabetes cases are attributed to type 2 diabetes mellitus (T2DM), with subsequent cardiovascular complications emerging as the primary drivers of morbidity and mortality. As a novel antidiabetic agent, glucagon-like peptide-1 receptor agonists (GLP-1 RAs) are seeing an increasing application in the management of patients with T2DM, given their remarkable efficacy in regulating blood sugar levels without posing an elevated risk of hypoglycemic episodes or weight gain (Drucker and Nauck, 2006; Nauck, 2016). Moreover, promising outcomes from various large-scale cardiovascular outcome trials (CVOTs) have indicated that GLP-1RAs could mitigate the risk of major adverse cardiovascular events (MACE) in T2DM patients with an elevated cardiovascular risk profile (Marso et al., 2016a; Marso et al., 2016b; Hernandez et al., 2018; Pfeffer et al., 2015; Holman et al., 2017; Husain et al., 2019; Gerstein et al., 2019). Due to these favorable attributes, GLP-1RAs have garnered endorsement from authoritative guidelines (Marx et al., 2023; 2024) as a significant therapeutic option for individuals with T2DM, especially those with preexisting atherosclerotic cardiovascular diseases or at a heightened cardiovascular risk.

However, safety concerns have persisted for years regarding the pancreatic effects of GLP-1 RAs. Based on observational data, a 2011 report highlighted an increased risk of pancreatitis and pancreatic cancer in patients using incretin therapy (Elashoff et al., 2011), prompting a warning from the Food and Drug Administration (FDA) regarding the pancreatic safety of GLP-1 RAs (Administration, 2013). A review of case reports (Franks et al., 2012) further heightened concerns about the potential adverse effects of GLP-1RAs on the pancreas, resulting in elevated pancreatic enzymes and AP. A meta-analysis of large randomized controlled trials examining the association between incretin-based therapies and AP revealed an 82% (95% CI, 1.17–2.82) higher likelihood of developing AP when using these drugs compared to conventional therapy (Roshanov and Dennis, 2015). While several recently published meta-analyses of CVOTs have shown that no such association was observed between GLP-1RAs and pancreatitis (Singh et al., 2020; Cao et al., 2020). Nevertheless, significant shortcomings existed in such studies, including relatively short mean follow-up times (of less than 2 years in the RCTs), selected patient cohorts, and limited sample sizes.

In this study, we conducted a review of published literature and an analysis of the US Food and Drug Administration Adverse Event Reporting System (FAERS) data to investigate the incidence of AP undergoing GLP-1 RAs. Our aim was to provide a comprehensive clinical depiction of AP induced by GLP-1 RAs and ascertain the presence of a safety signal between AP and GLP-1 RAs in real-world settings.

## 2 Methods

### 2.1 Case series

We conducted a comprehensive literature search using Google Scholar, Scopus, PubMed, and Web of Science, focusing on English-language publications up to 31 December 2023. The following search terms were used: (exenatide OR liraglutide OR albiglutide OR dulaglutide OR lixisenatide OR semaglutide OR tirzepatide OR GLP-1 RAs OR Glucagon-like peptide-1 receptor agonist) AND (acute pancreatitis) AND (case report OR case series). The eligibility criteria included any case report or case series that documented instances of AP during the administration of GLP-1 RAs. Patient demographics such as age and gender, along with dosage, treatment duration, presenting symptoms, imaging results, causality assessment (using Naranjo scale) (Naranjo et al., 1981), acute pancreatitis management, and outcomes were extracted from the examination of medical files.

### 2.2 Phamacovigilance analysis

This retrospective pharmacovigilance analysis is based on real-world data sourced from individual case safety reports (ICSRs) submitted to the FAERS. FAERS compiles information on adverse events, medication errors, and product quality complaints leading to adverse events. It serves as a cornerstone of the FDA’s post-marketing safety surveillance initiative for pharmaceuticals and therapeutic agents, operating as a classic spontaneous reporting system. The database captures a wide array of data including demographics, drug details, indications, outcomes, adverse reactions, sources, and therapies. Data submitting to ICSRs with GLP-1 RAs as suspected drugs were extracted from the FAERS database spanning the period between January 2005 and September 2023. Utilizing the Medical Dictionary for Regulatory Activities (MedDRA) version 26.0, we identified 25 preferred terms (PTs) (Supplementary Table S1) to gather pertinent cases linked to “acute pancreatitis” (Standardized MedDRA Queries (SMQ): 20000022) and closely related clinical conditions. To ensure data integrity, we conducted a thorough review to eliminate potential duplicates, defined as records sharing at least three out of four key fields: event date, age, sex, and reporter’s country. Additionally, incorrect data were excluded, such as cases where the GLP-1 RA initiation date was later than the onset date of pancreatitis.

### 2.3 Statistical analysis

The clinical profile, such as age, sex, primary data source, outcomes, reported year, source region, and indication, were detailed individually for each GLP-1 RAs. Disproportionality analysis and Bayesian analysis were employed, utilizing the reporting odds ratio (ROR), proportional reporting ratio (PRR), Bayesian confidence propagation neural network (BCPNN), and multi-item gamma Poisson shrinker (MGPS) algorithms to identify associations between different GLP-1 RAs and AP events. The equations and criteria for these algorithms (Chen et al., 2020) are detailed in Supplementary Table S2. If any of the four algorithms met the predefined criteria, a positive signal of AP was identified. Analyses were performed using SPSS 23.0 (IBM, Armonk, NY, United States) statistical software.

## 3 Results

### 3.1 Case series

During the study period, thirty-nine patients experienced new-onset AP while using GLP-1 RAs (Table 1). More specifically, among these cases, 19 (48.7%) were associated with liraglutide, 9 (23.1%) with dulaglutide, 4 (10.3%) each with exenatide and semaglutide, while 1 (2.6%) case each was linked to lixisenatide, albiglutide, and tirzepatide. The median age at the onset of AP was 60 years (range: 27–77 years), with 20 (51.8%) being male. All patients in the study were identified as having either type T2DM or obesity, with the exception of one patient who had been diagnosed with prediabetes. Notably, 10 cases (25.6%) involved an escalation in drug dosage within 3 months preceding the event. The median time to onset was 2.5 months (range 0 days–3 years). The predominant presenting symptom was epigastric abdominal pain accompanied by nausea and vomiting. Most cases exhibited evidence of pancreatitis on CT scans. Using the Naranjo scale, 33 cases (84.6%) were deemed to have a probable causal relationship between GLP-1 RAs and AP, while 4 cases (10.3%) were classified as possible. None of the patients from the case series was rechallenged with GLP-1 RAs due to safety concerns. Two cases involved cholelithiasis, and two patients received treatment with either empagliflozin or sitagliptin, which could potentially contribute to or confound pancreatitis. The standard management approach for these patients involved discontinuation of GLP-1 RAs and supportive care, including intravenous fluids and pain management. The majority of patients recovered without complications following this treatment regimen, except for one patient who experienced a fatal outcome.

**TABLE 1:** Summary of case reports of GLP-1 receptor agonists-induced acute pancreatitis reported in the literature.

References	Country	Age (year) and gender	Indication	Medication	Dose	Duration	Complaints	Imaging findings	Naranjo result	Treatment	Outcome
Denker and Dimarco (2006)	United States	69 M	T2DM	Exenatide	5 mg bid	Within 24 h	Midepigastric abdominal pain radiating to the back	CT: no evidence of cholelithiasis	Probable	Discontinued exenatide, antoprazole and IV fluids	Recovered
Tripathy et al. (2008)	India	52 F	T2DM	Exenatide	5 mg bid	1 day	Abdominal pain, nausea, vomiting and fever	Ultrasound: significant abnormality	Probable	Discontinued exenatide, NPO, intensive antibiotic therapy, IV fluids	Recovered
Ayoub et al. (2010)	United States	64 F	T2DM	Exenatide	5 mg bid	2 days	Epigastric pain aggravated by food	CT: a enlarged pancreas, particularly at the head and body, with surrounding edema	Probable	Discontinued exenatide, NPO, IV fluids, pain medications and pantoprazole	Recovered
Iyer et al. (2012)	United States	76 F	T2DM	Exenatide and sitagliptin	5 mg qd	3 years	Severe abdominal pain, vomiting, and fever	CT: generalized peripancreatic stranding and dissecting fluid, then developed into extensive pancreatic parenchymal necrosis with a large amount of gas tracking throughout the pancreatic band	Possible	Discontinued exenatide and sitagliptin, supportive care	Died
Lee et al. (2011)	United States	60 F	T2DM	Exenatide 10 µg bid for approximately 4 years and then switched to liraglutide	Liraglutide 1.8 mg qd	23 days	Midepigastric pain radiating to the back	CT: pancreatic calcification	Probable	Discontinued liraglutide and IV fluids	Recovered
Bourezane et al. (2012)	France	63 M	T2DM	Liraglutide	0.6 mg and gradually increased to 1.8 mg qd for 1 month	330 days	Midepigastric pain radiating to the back, flank, chest and lower abdomen	CT: infiltration of peripancreatic fat and presence of fluid collections	Probable	Discontinued liraglutide, insulin, IV fluids and analgesics	Improved
Knezevich et al. (2012)	United States	53 M	T2DM	Liraglutide	Increased from 0.6 to 1.2 mg qd	2 months	Intolerable abdominal pain in the right upper quadrant and left upper quadrant	CT: peripancreatic inflammation	Probable	Discontinued all oral medications, IV fluids and analgesics	Recovered
Taunk et al. (2012)	United States	74 M	T2DM	Liraglutide	0.6 mg bid	1 month	Abdominal pain and vomiting	—	—	Discontinued liraglutide	Recovered
Nakata et al. (2012)	Japan	75 F	T2DM	Liraglutide	0.6 mg qd	9 months	Nausea	CT: swelling of the pancreatic tail	Probable	Discontinued liraglutide	Recovered
Famularo et al. (2012)	Italy	67 M	T2DM	Liraglutide	1.2 mg qd	5 months	Nausea, vomiting, and constant pain in the epigastrium	MRI: a moderately enlarged and edematous pancreas	Probable	Discontinued liraglutide and IV fluids	Recovered
Jeyaraj et al. (2014)	India	51 F	T2DM	Liraglutide	0.6 mg for 1 week and increased to 1.2 mg for 7 weeks	8 weeks	Severe abdominal pain, nausea and vomiting	CT: mild enlargement of the pancreas with reduced parenchymal enhancement	Probable	Discontinued liraglutide, antibiotics, IV fluids and insulin	Recovered
Ghabra and Alkhouli (2018)	United States	27 F	T2DM	Liraglutide	—	2 weeks	Epigastric pain radiating into the back, diarrhea	—	—	Discontinued liraglutide, antiemetics, IV fluids and analgesics	Improved
Quesada-Vázquez (2018)	UAE	44 F	Obesity	Liraglutide	1.2 mg qd	6 months	Epigastric pain radiating to the back	—	Probable	Discontinued liraglutide	Recovered
Farooqui et al. (2019)	Qatar	64 F	T2DM	Liraglutide	—	4 weeks	Epigastric pain, nausea	MRI: no significant pathology or obstruction	Probable	Discontinued liraglutide	Recovered
Al-Salameh et al. (2019)	United States	53 F	T2DM	Liraglutide	1.2 mg and increased to 1.8 mg qd for 2 days	—	Epigastric abdominal pain, nausea, and non-bilious emesis	Ultrasound and CT: no evidence of biliary pathology	Probable	Discontinued liraglutide and supportive care	Recovered
Fatakhova et al. (2019)	United States	40 F	Obesity	Liraglutide	—	4 weeks	Sharp epigastric pain radiating to the back, nausea	CT: cholelithiasis without evidence of cholecystitis	Possible	-	Improved
Gameil and Elsebaie (2020)	Egypt	53 M	T2DM	Liraglutide	0.6 mg increased to 1.2 mg and later 1.8 mg qd	3 months	Mild abdominal discomfort and repeated vomiting	CT: a diffuse enlarged pancreas with heterogeneous enhancement of the parenchyma, irregular contour with peripancreatic edema, and fat strands	Probable	Discontinued liraglutide, soft enteral feeding, antibiotics and insulin	Recovered
Dolan et al. (2020)	United States	31 F	T2DM	Liraglutide	3 mg qd	10 months	Sharp midepigastric pain radiating to the back and left upper abdomen	CT: mild interstitial pancreatitis	Probable	Discontinued liraglutide, pain management, fluid resuscitation, and early enteral feeds	Improved
Chua and Ng (2021)	Singapore	57 F	T2DM	Liraglutide	0.6 mg	5 days	Abdominal pain in the epigastric region, nausea and vomiting	CT: peripancreatic fluid and fat stranding around the tail of the pancreas	Probable	Discontinued liraglutide, analgesics, IV fluids and soft diet.	Recovered
Fernandez et al. (2021)	United States	48 M	T2DM	Liraglutide and empagliflozin	-	2 months	Acute abdominal pain, nausea and vomiting	CT: large peripancreatic fluid collection	Possible	Discontinued liraglutide, IV fluids and antibiotics	Improved
AlSaadoun et al. (2022)	SAU	25 F	Obesity	Liraglutide	2.4 mg	2 months	Sharp epigastric abdominal pain, nausea and non-bloody, nonbilious emesis	Ultrasound: negative for cholelithiasis, cholecystitis, or biliary ductal dilatation	Probable	Discontinued liraglutide, bowel rest, analgesics, IV fluids, antibiotics, and clexane	Improved
Easow et al. (2022)	India	69 M	T2DM	Liraglutide	1.2 mg	3 years	Abdominal pain in the epigastric region and vomiting	MRI: a stone of 8 mm in the ampulla of Vater producing dilation of the pancreatic duct	Possible	Discontinued liraglutide	Improved
Javed et al. (2023)	United States	73 M	T2DM	Liraglutide	—	20 months	Abdominal pain in the epigastric region, dry heaves and subjective fevers	CT: diffuse edematous inflammation of pancreatic head, body, and tail	Probable	Discontinued liraglutide and IV fluids	Recovered
Jain et al. (2016)	United States	59 M	T2DM	Albiglutide	30 mg qw	26 days	Epigastric pain, nausea	CT: no pancreatic findings	Probable	Discontinued albiglutide, IV fluids, pain medications and insulin	Improved
Bhat and Goudarzi (2021)	United States	69 M	T2DM	Dulaglutide	0.75 mg and increased to 1.5 mg qw for 3 days	3 months	Diffuse abdominal pain, nausea and vomiting	CT: an enlarged pancreas with peripancreatic stranding and slightly diminished enhancement	Probable	Meropenem for necrotizing pancreatitis	Recovered
Cheng et al. (2021)	United States	61 M	T2DM	Dulaglutide	1.5 mg qw	5 months	Acute epigastric pain	Ultrasound: no cholelithiasis, no acute cholecystitis	Probable	Discontinued dulaglutide, IV fluids and analgesics	Recovered
Abdelmasih et al. (2022)	United States	77 M	T2DM	Dulaglutide	1.5 mg and increased to 3 mg qw for 2 weeks	-	Epigastric pain, nausea, and vomiting	CT: confirmed pancreatitis	Probable	Discontinued dulaglutide	Recovered
Babajide et al. (2022)	United States	61 M	T2DM	Dulaglutide	0.75 mg qw	6 months	Upper abdominal pain, nausea and vomiting	CT: increased peripancreatic fat stranding, fluid	Probable	Discontinued dulaglutide and IV fluids	Recovered
Yau et al. (2022)	United States	46 M	T2DM	Dulaglutide	—	—	Severe right upper quadrant abdominal pain, nausea	CT: focal hypoattenuation/edema of the pancreatic head with surrounding fat stranding	Probable	Discontinued dulaglutide and IV fluids	Recovered
Khan et al. (2023)	PAK	37 M	T2DM	Dulaglutide	0.75 mg and increased to 1.5 mg qw for 2 weeks	—	Abdominal pain, nausea and vomiting	CT: fat stranding around the pancreas	Probable	IV fluids and as-needed pain medication	Improved
Shahbazi et al. (2023)	United States	56 M	T2DM	Dulaglutide	0.75 mg and increased to 1.5 mg recently	4 weeks	Abdominal pain and nausea	CT: extensive interstitial edema around the pancreatic tail along with peripancreatic fat stranding	Probable	Discontinued dulaglutide, IV fluids, rectal bisacodyl, and linaclotide	Improved
Manuel et al. (2023)	United States	57 M	T2DM	Dulaglutide	1.5 mg and increased to 3 mg qw for 3 months	2 years	Abdominal pain	CT: a hazy inflammatory stranding around the pancreatic uncinate process	Probable	Discontinued dulaglutide, IV fluids and pain management	Improved
Kumar Kulkarni et al. (2023)	United States	68 F	T2DM	Dulaglutide	—	4 years	Severe epigastric pain, nausea and vomiting	CT: acute pancreatitis and no gallstones or bile duct dilatation	Probable	Discontinued dulaglutide, IV fluids, NPO diet and morphine	Improved
Chis and Fodor (2018)	Romania	67 M	T2DM	Lixisenatide	10 mg qd	3 months	Intense epigastric pain, nausea and vomiting	CT: the peripancreatic fatty tissue and pancreatic edema	Probable	Discontinued lixisenatide, IV fluids, proton pump inhibitor and antispasmodic drugs	Recovered
Nohomovich et al. (2023)	United States	60 + F	T2DM	Semaglutide	—	6 weeks	Abdominal pain	CT: enlargement of the pseudocyst to approximately 7 cm in size with ascites	Probable	Discontinued semaglutide, ampicillin-sulbactam and surgery to drain the pseudocyst	Improved
Patel et al. (2023)	United States	61 F	T2DM	Semaglutide	0.5 mg qw	-	Sudden onset abdominal pain	CT: no acute abnormality	Probable	Discontinued semaglutide	Recovered
Ebiai et al. (2023)	United States	60 F	T2DM	Semaglutide	0.5 mg qw for 24 months and increased to 1.0 mg qw for 3 weeks	24 months	Severe abdominal pain, nausea and vomiting	CT: pancreatic fat stranding	Probable	—	—
Kumar Kulkarni et al. (2023)	United States	50 M	T2DM	Semaglutide	—	6 months	Acute severe epigastric pain, nausea and vomiting	CT: acute interstitial edematous pancreatitis without cholelithiasis or choledocholithiasis	Probable	Discontinued semaglutide, IV fluids, NPO diet and morphine	Improved
Casanovas et al. (2023)	United States	38 F	Pre-diabetes	Tirzepatide	Increased to 7.5 mg for 1 day before symptoms appeared	2 months	Epigastric pain diarrhea, nausea and vomiting	CT: edematous changes in the pancreatic head and uncinate process region	Probable	Discontinued tirzepatide	Improved

Abbreviations: T2DM, type 2 diabetes mellitus; CT, computed tomography; IV, intravenous; NPO, nil per os; MRI, magnetic resonance imaging.

UAE: United Arab Emirates; SAU: Saudi Arabia; PAK: Pakistan.

### 3.2 Descriptive analysis from FAERS

**FIGURE 1:**
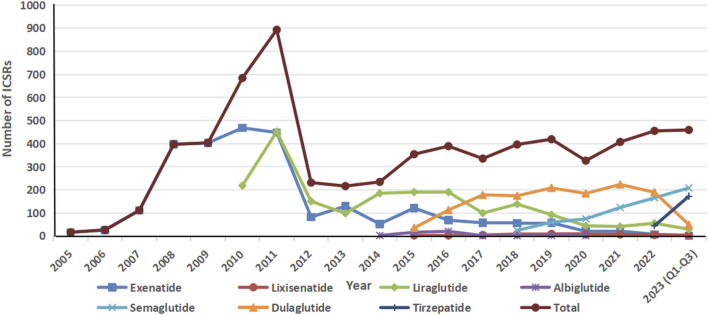
Distribution of Individual Safety Reports having GLP-1 RAs as suspect drugs by year (2005–2023 (Q1-Q3))

In total, the FAERS database archived 6,751 reports related to acute pancreatitis induced by GLP-1 RAs from January 2005 to September 2023. Specifically, 2,539 ICSRs (37.6%) were associated with exenatide, 1981 (29.3%) with liraglutide, and 1,352 (20.0%) with dulaglutide. The demographic and clinical characteristics of all ICSRs are outlined in Table 2. The median age of patients across all ICSRs was 57 years (range: 14–99, n = 2,815), similar to that of each specific GLP-1 RA. Female patients accounted for the highest proportion of ICSRs (45.8%), and 6,634 (98.3%) cases were classified as serious. The majority of reports (63.4%) were submitted by healthcare professionals and originated from North America (87.8%). In terms of outcomes, other adverse events (51.5%) were the most prevalent, followed by hospitalization (40.4%), life-threatening (2.8%) and death (2.7%). AP events were predominantly reported for unknown indications (53.8%) and T2DM (43.0%). The events manifested soon after the initiation of GLP-1 RA treatment, with a median onset time of 92 days (range: 0–3,312, n = 1,591) across all ICSRs that provided both drug initiation and AP onset times. Notably, 30.9% of these reports were gathered within the initial month, and almost half (48.5%) were compiled within the first 3 months after eliminating invalid reports. The number of acute pancreatitis adverse events steadily increased from 16 in 2005 to 459 in 2023 (Q1-Q3), peaking in 2011, reflecting the growing clinical utilization of GLP-1 RAs (Figure 1).

**TABLE 2:** Clinical characteristics of patients with GLP-1 receptor agonists-associated acute pancreatitis collected from the FAERS database (January 2005 to September 2023).

Variables	Exenatide n = 2,539	Lixisenatide n = 45	Liraglutide n = 1,981	Albiglutide n = 43	Semaglutide n = 653	Dulaglutide n = 1,352	Tirzepatide n=216	Total n = 6,751
Age median (range)	57 (18–99) n = 955	58.5 (26–79) n = 24	56 (14–96) n = 1,058	59 (35–79) n = 15	59 (17–83) n = 335	58 (20–88) n = 561	52 (21–82) n = 79	57 (14–99) n = 2,815
Sex								
Male	1,192 (46.9)	13 (28.9)	739 (37.3)	20 (46.5)	305 (46.7)	549 (40.6)	49 (22.7)	2,837 (42.0)
Female	1,253 (49.4)	16 (35.6)	917 (46.3)	17 (39.5)	307 (47.0)	523 (38.7)	105 (48.6)	3,095 (45.8)
Not reported	94 (3.7)	16 (35.6)	325 (16.4)	6 (14.0)	41 (6.3)	280 (20.7)	62 (28.7)	819 (12.1)
Primary source								
Healthcare professional	1,295 (51.0)	36 (80.0)	1,690 (85.3)	40 (93.0)	529 (81.0)	716 (53.0)	23 (10.6)	4,281 (63.4)
Consumer	1,223 (48.2)	8 (17.8)	268 (13.5)	3 (7.0)	120 (18.4)	634 (46.9)	193 (89.4)	2,419 (35.8)
Not specified	21 (0.8)	1 (2.2)	23 (1.2)	—	4 (0.6)	2 (0.1)	—	51 (0.8)
Outcomes								
Non-serious	41 (1.6)	1 (2.2)	34 (1.7)	1 (2.3)	12 (1.8)	24 (1.8)	1 (0.5)	114 (1.7)
Hospitalization	998 (39.3)	21 (46.7)	864 (43.6)	20 (46.5)	240 (36.8)	539 (39.9)	69 (31.9)	2,730 (40.4)
Disability	41 (1.6)	1 (2.2)	11 (0.6)	2 (4.7)	2 (0.3)	5 (0.4)	—	62 (0.9)
Life-threatening	84 (3.3)	1 (2.2)	55 (2.8)	—	19 (2.9)	34 (2.5)	—	192 (2.8)
Death	101 (4.0)	—	44 (2.2)	3 (7.0)	17 (2.6)	25 (1.8)	2 (0.9)	179 (2.7)
Other	1,274 (50.2)	21 (46.7)	973 (49.1)	17 (39.5)	363 (55.6)	725 (53.6)	144 (66.7)	3,474 (51.5)
Source region								
Africa	6 (0.2)	—	1 (0.1)	—	—	2 (0.1)	—	9 (0.1)
Asia	41 (1.6)	7 (15.6)	34 (1.7)	—	7 (1.1)	22 (1.6)	5 (2.3)	106 (1.6)
Europe	229 (9.0)	11 (24.4)	191 (9.6)	—	60 (9.2)	90 (6.7)	—	582 (8.6)
North America	2,195 (86.5)	25 (55.6)	1713 (86.5)	41 (95.3)	576 (88.2)	1,231 (91.1)	211 (97.7)	5,927 (87.8)
Oceania	46 (1.8)	—	6 (0.3)	—	5 (0.8)	1 (0.1)	—	56 (0.8)
South America	15 (0.6)	2 (4.4)	33 (1.7)	—	5 (0.8)	5 (0.4)	—	59 (0.9)
Country not specified	7 (0.3)	—	3 (0.2)	2 (4.7)	—	1 (0.1)	—	12 (0.2)
Indication								
Diabetes mellitus	1,039 (40.9)	17 (37.8)	1,061 (53.6)	20 (46.5)	199 (30.5)	531 (39.3)	70 (32.4)	2,903 (43.0)
Other	16 (0.6)	1 (2.2)	129 (6.5)	-	38 (5.8)	10 (0.7)	13 (6.0)	218 (3.2)
Unknown	1,484 (58.4)	27 (60.0)	791 (39.9)	23 (53.5)	416 (63.7)	811 (60.0)	133 (61.6)	3,630 (53.8)
Time to onset median (range), days	212 (0–2,642) n = 676	12 (8–16) n = 2	81 (0–3,312) n = 522	48 (0–434) n = 6	45 (0–1829) n = 179	45 (0–1829) n = 179	31 (0–516) n = 43	92 (0–3,312) n = 1,591

### 3.3 Signal values associated with different GLP-1 RAs

We identified signals of AP events associated with all GLP-1 RAs using the criteria established by the four algorithms, and the results are summarized in Table 3. Each GLP-1 RA satisfied all four criteria, as did the overall group of GLP-1 RAs. Notably, among all GLP-1 RAs, liraglutide stood out for its association with AP events related to acute pancreatitis. This is highlighted by its notably highest values across various statistical parameters, including an IC at 4.17 (IC025 3.98), an ROR at 20.13 (95% CI 19.21–21.09), and an EBGM at 18.04 (EBGM05 17.35). Following liraglutide, exenatide, semaglutide, dulaglutide, lixisenatide, and albiglutide exhibited progressively lower values, while tirzepatide demonstrated the lowest association.

**TABLE 3:** Associations of GLP-1 receptor agonists with acute pancreatitis.

GLP-1 RAs	N	ROR (95% CI)	PRR (χ^2^)	IC (IC025)	EBGM (EBGM05)
Exenatide	2,539	13.00 (12.48, 13.54)	12.48 (25294.99)	3.56 (3.42)	11.79 (11.39)
Lixisenatide	45	5.83 (4.34, 7.83)	5.73 (176.08)	2.52 (1.87)	5.72 (4.47)
Liraglutide	1,981	20.13 (19.21, 21.09)	18.88 (32075.86)	4.17 (3.98)	18.04 (17.35)
Albiglutide	43	4.73 (3.50, 6.39)	4.67 (124.18)	2.22 (1.64)	4.66 (3.62)
Semaglutide	653	8.23 (7.61, 8.90)	8.02 (3,966.43)	2.98 (2.76)	7.91 (7.41)
Dulaglutide	1,352	6.69 (6.34, 7.07)	6.56 (6,192.43)	2.67 (2.53)	6.38 (6.10)
Tirzepatide	216	2.94 (2.57, 3.36)	2.92 (272.29)	1.54 (1.35)	2.91 (2.60)
Total	6,751	11.62 (11.32, 11.93)	11.25 (53134.84)	3.26 (3.18)	9.61 (9.40)

Abbreviations: GLP-1, RAs; GLP-1, receptor agonists; N, the number of reports of GLP-1, RAs associated-acute pancreatitis; ROR, reporting odds ratio; CI, confidence interval; PRR, proportional reporting ratio; χ^2^, chi-squared; IC, information component; EBGM, empirical Bayes geometric mean.

We further examined adverse events related to AP at the PT level and listed all signal-based ROR criteria in Table 4. As depicted in Table 4, exenatide exhibited the broadest spectrum, with a total of 8 potential signals indicating GLP-1 RA-induced AP, ranging from pancreatic abscess (ROR 4.20, 95% CI 1.03–17.03) to pancreatic phlegmon (ROR 84.53, 95% CI 21.86–326.90). Conversely, lixisenatide, albiglutide, and tirzepatide showed the fewest PTs, with only two signals detected for each drug. Among all ICSRs, cases involving pancreatitis and acute pancreatitis were the most frequently reported PTs for all drugs.

**TABLE 4:** Signal strength for GLP-1 receptor agonists based on PT level in FAERS.

PT	Exenatide		Lixisenatide		Liraglutide		Albiglutide		Semaglutide		Dulaglutide		Tirzepatide	
	N	ROR (95% CI)	N	ROR (95% CI)	N	ROR (95% CI)	N	ROR (95% CI)	N	ROR (95% CI)	N	ROR (95% CI)	N	ROR (95% CI)
Oedematous pancreatitis	—		—		3	4.58 (1.47, 14.29)	—		4	8.16 (3.04, 21.92)	4	3.17 (1.18, 8.52)	—	
Pancreatic abscess	2	4.20 (1.03, 17.03)	—		—		—		—		—		—	
Pancreatic phlegmon	3	84.53 (21.86, 326.90)	—		—		—		—		—		—	
Pancreatic pseudocyst	19	10.80 (6.81, 17.14)	—		13	14.28 (8.21, 24.84)	—		—		—		—	
Pancreatitis	1963	14.03 (13.39, 14.70)	28	5.05 (3.48, 7.33)	1,525	21.48 (20.38, 22.64)	33	5.08 (3.60, 7.16)	545	9.62 (8.83, 10.48)	1,151	8.02 (7.56, 8.51)	190	3.63 (3.15, 4.19)
Pancreatitis acute	577	10.93 (10.05, 11.89)	17	8.40 (5.21, 13.54)	481	17.86 (16.29, 19.58)	10	4.19 (2.25, 7.80)	90	4.24 (3.45, 5.22)	184	3.39 (2.93, 3.93)	—	
Pancreatitis haemorrhagic	19	15.24 (9.55, 24.30)	—		—		—		—		—		—	
Pancreatitis necrotising	51	8.51 (6.43, 11.26)	—		28	9.01 (6.19, 13.10)	—		17	7.22 (4.47, 11.65)	16	2.64 (1.64, 4.32)	6	2.81 (1.26, 6.26)
Pancreatitis relapsing	23	13.47 (8.83, 20.54)	—		—		—		—		—		—	
Total	2,657		45		2050		43		656		1,355		196	

Abbreviations: PT, preferred term.

## 4 Discussion

In conclusion, we found significant over-representation of signals for acute pancreatitis (SMQ: 20000022) over other adverse reactions for all GLP-1 RAs. Though the disproportionality analysis and Bayesian analysis as a rapid and effective method for signal detection, our study represents the largest post-marketing surveillance to date of these GLP-1 RAs. We have provided valuable and timely evidence for clinical evaluation, aiming to mitigate the potential harm associated with acute pancreatitis following treatment with GLP-1 RAs.

Overall, from the first quarter of 2005 to the third quarter of 2023, there were 6,751 reports describing acute pancreatitis associated with GLP-1 RAs in the FAERS database. Both the pharmacovigilance findings and the case series indicated that liraglutide and dulaglutide were the leading suspected GLP-1 RAs, and pharmacovigilance analysis showed that exenatide had the highest number of ICSRs associated with AP. The median age of patients was 57 years (range: 14–99 years) in our pharmacovigilance analysis and 60 years (range: 27–77 years) for the cases of GLP-1 RAs-induced AP published in the case reports, which is in line with earlier observational studies on drug-induced AP (Gagnon et al., 2020; Chadalavada et al., 2020). Our pharmacovigilance results suggest that AP associated with GLP-1 RAs was more frequently reported in females, while the case series results did not show the same trend. However, the validity of this finding cannot be conclusively confirmed, given the multitude of factors that can influence the spontaneous reporting of adverse events. Additionally, the gender of 12.1% of the ICSRs was not reported, which further complicates the analysis. Nevertheless, there is some evidence suggesting that females may experience this condition more frequently (Barreto et al., 2011; Kaufman, 2013). We also observed that the median time to onset of GLP-1 RAs-associated acute pancreatitis was 92 (range: 0–3,312) days across ICSRs that provided both drug initiation and AP onset times, and 2.5 months of the case series, indicating a longer onset duration compared to other gastrointestinal adverse events triggered by GLP-1 RAs (Zhou et al., 2022).

In our study, excluding the initial 3 years since the launch of exenatide, the reported cases have averaged nearly 400 per year since 2015. However, there was a notable surge in cases during 2010 and 2011, with 684 cases reported in 2020 and 893 cases in 2021. This surge may be attributed to the FDA mandating manufacturers of incretin-based medications to revise their product labels in 2009, providing information regarding the potential risk of pancreatitis (Nelson et al., 2014). Approximately 87.8% of the reports were derived from North America, which may be attributed to FAERS being established in the United States. Furthermore, 40.4% of ICSRs involved hospitalized patients, 2.7% resulted in patient mortality, 0.9% led to disability, and 2.8% caused life-threatening reactions, while only 1.7% classified as non-serious outcomes. Additionally, within the case series results, one patient (2.6%) died, while 2.7% of ICSRs from our FAERS analysis had a fatal outcome, underscoring the seriousness of acute pancreatitis and the necessity for specialized attention.

Our study detected a notable signal between different GLP-1 RAs and AP in the FAERS database throughout the study duration. Meanwhile, liraglutide exhibited the strongest association with acute pancreatitis, evidenced by the highest values of IC, ROR, and EBGM. Following liraglutide, exenatide emerged as the second-highest in terms of this association. Despite exenatide showing a higher reported number of reactions compared to liraglutide (2539:1981), the associations with acute pancreatitis events were weaker, which was also observed in cases of pancreatic cancer (Cao et al., 2023). It is suggested that patients at risk of pancreatitis avoid using any GLP-1 RAs, particularly liraglutide and exenatide. And the association of tirzepatide with acute pancreatitis events appears to be the weakest, possibly due to its later launch on the market.

AP ranks as the primary cause of hospital admissions for gastrointestinal disease (Mossad et al., 2017) and the fifth leading cause of in-hospital mortality in the United States (Sorribas et al., 2023). Addressing the underlying causes of pancreatitis is essential to prevent its recurrence. Gallstones and alcohol abuse stand out as the primary triggers for AP, while genetic factors, medications, and smoking also play contributing roles (Lee and Papachristou, 2019). Additionally, T2DM poses a significant risk for AP, particularly among younger diabetic patients (Lankisch et al., 2015). Moreover, worsening glycemic control escalates the likelihood of AP (Cho et al., 2023). Although drugs only account for 0.1%–2% of AP cases, their impact can be life-threatening (Wolfe et al., 2020). Therefore, managing drug-induced AP necessitates discontinuing the causative medication and providing supportive care. The GLP-1RAs should not be restarted if pancreatitis is confirmed (Wharton et al., 2022), and none of the patients from the case series were rechallenged with GLP-1 RAs for safety reasons. Failure to identify the responsible drug can lead to significant delays in treatment, potentially resulting in critical outcomes (Jones et al., 2015). Unraveling a causal relationship between GLP-1 agonists and AP is intricate, particularly as patients with T2DM are already three times more predisposed to pancreatitis compared to their non-diabetic counterparts (Girman et al., 2010). Therefore, it's imperative to examine all plausible factors and to rely on a diagnosis of exclusion when attributing AP to drug-induced causes.

Three out of thirty-nine patients (7.7%) from the case series were diagnosed with obesity. Obesity doesn't just pose a risk for local and systemic complications in acute pancreatitis; it also elevates mortality rates associated with this condition (Martínez et al., 2006). Currently, the FDA has approved three GLP-1 RAs for obesity treatment: liraglutide, semaglutide and tirzepatide. Notably, the dosage for obesity treatment is considerably higher than that for diabetes management. Take semaglutide as an example; the maintenance dose for the treatment of obesity is 2.4 mg subcutaneously once a week, whereas for diabetes, the maximum dose is 1 mg subcutaneously once a week. Whether this elevated dosage could potentially increase the risk of acute pancreatitis in obese patients compared to those with diabetes is a subject that necessitates further investigation.

In this study, we applied four algorithms to analyze the association between GLP-1 RAs and acute pancreatitis. Each method has distinct advantages and limitations. BCPNN and MGPS are Bayesian approaches known for their higher specificity (Bate et al., 1998; DuMouchel, 1999). They are particularly useful when working with sparse data or for pattern recognition in higher dimensions, making them applicable in a variety of scenarios. However, they are less sensitive compared to frequentist methods and can be less transparent to those unfamiliar with Bayesian statistics (Almenoff et al., 2006). On the other hand, PRR and ROR are frequentist approaches that are simpler to apply and interpret (Evans et al., 2001; van Puijenbroek et al., 2002). They have the advantage of higher sensitivity, making them useful for early detection of adverse drug events (Li et al., 2008). However, these methods are less specific and can sometimes produce false positives, particularly in rare drug-event combinations. The consistency of signals across all four methods strengthens our findings and minimizes the influence of biases inherent to any single algorithm. The convergence of these results enhances confidence in the association between GLP-1 RAs and acute pancreatitis, ensuring a comprehensive and reliable evaluation of the data.

Additionally, clinicians should view the statistical associations observed in this study as hypothesis-generating rather than conclusive evidence of a cause-and-effect relationship. The primary metrics used in this study, including the reporting odds ratio (ROR) and Bayesian confidence propagation neural network (BCPNN) indicators, are designed to identify disproportionalities in reporting patterns. These tools help detect potential safety signals but do not account for confounding variables such as baseline patient characteristics, comorbidities, or concomitant medication use. Consequently, the presence of a signal should be interpreted as an indication of potential risk that needs to be further evaluated in the context of robust, well-controlled clinical studies.

Despite the advantages of real-world studies and data mining techniques in this research, there are numerous limitations to consider. Firstly, the spontaneous reporting system is affected by limitations within the FAERS database, including duplicate reports, reporting accuracy and quality, incomplete or insufficient details regarding drug administration (such as site, route, dose and timing), and the lack of important patient characteristics (such as medical history and comorbidities). Secondly, reports from FAERS lack medical confirmation, potentially introducing reporter bias (Nomura et al., 2015). As a result, data mining alone does not provide sufficient evidence to establish causality and primarily emphasizes the need for practitioner vigilance. It is important to note that all signal detection can only suggest a statistical correlation, and further investigation and research are needed to determine if there is a real causal relationship. Lastly, despite individually reviewing ICSRs in our study and considering data on other drugs that could potentially induce adverse reactions, the possibility of notoriety bias cannot be dismissed. Despite these inherent limitations in spontaneous reporting, the FAERS database remains a valuable resource. Data mining remains a critical tool for the ongoing assessment and management of risks associated with commercially available pharmaceutical products.

## 5 Conclusion

In conclusion, a notable reporting signal for acute pancreatitis exists across all GLP-1 RAs in the FAERS database, particularly associated with exenatide and liraglutide. Clinicians must be vigilant and monitor this potentially serious adverse event. Moreover, we anticipate further pharmacovigilance studies, cohort analyses, and clinical trials in the future to develop evidence-based treatment strategies for patients experiencing GLP-1 RA-induced AP.

## Data Availability

The original contributions presented in the study are included in the article/Supplementary Material, further inquiries can be directed to the corresponding author.
