# Supplementary material for: Association between different GLP-1 receptor agonists and acute pancreatitis: case series and real-world pharmacovigilance analysis
Source: Front Pharmacol. 2024 Nov 13;15:1461398. doi: 10.3389/fphar.2024.1461398 (PMC11600108; doi:10.3389/fphar.2024.1461398)
Supplement: Supplementary file 1 [file Table1.DOCX]

Supplementary Material

Table S1 PTs contained in the narrow-scope search of “Acute pancreatitis (SMQ)”

| PT | MedDRA code |
| --- | --- |
| Cullen's sign | 10059029 |
| Grey Turner's sign | 10075426 |
| Haemorrhagic necrotic pancreatitis | 10076058 |
| Hereditary pancreatitis | 10056976 |
| Idiopathic pancreatitis | 10088882 |
| Immune-mediated pancreatitis | 10083072 |
| Ischaemic pancreatitis | 10066127 |
| Oedematous pancreatitis | 10052400 |
| Pancreatic abscess | 10048984 |
| Pancreatic cyst drainage | 10082531 |
| Pancreatic haemorrhage | 10033625 |
| Pancreatic phlegmon | 10056975 |
| Pancreatic pseudoaneurysm | 10081762 |
| Pancreatic pseudocyst | 10033635 |
| Pancreatic pseudocyst drainage | 10033636 |
| Pancreatic pseudocyst haemorrhage | 10083813 |
| Pancreatic pseudocyst rupture | 10083811 |
| Pancreatitis | 10033645 |
| Pancreatitis acute | 10033647 |
| Pancreatitis haemorrhagic | 10033650 |
| Pancreatitis necrotising | 10033654 |
| Pancreatitis relapsing | 10033657 |
| Pancreatorenal syndrome | 10056277 |
| Subacute pancreatitis | 10084554 |
| Walled-off pancreatic necrosis | 10085347 |

Abbreviations PT, preferred term; MedDRA, Medical Dictionary for Drug Regulatory Activities; SMQ, Standardized MedDRA Querie.

Table S2 Summary of major algorithms used for signal detection

| Algorithms | Equation | Criteria |
| --- | --- | --- |
| ROR | ROR=ad/bc  95%CI=e^ln(ROR)^±1.96(1/a+1/b+1/c+1/d)^0.5 | 95%CI>1, N≥2 |
| PRR | PRR = a(c + d)/c/(a + b)  χ^2^ = [(ad−bc)^2^](a + b + c + d)/[(a + b)(c + d)(a + c)(b + d)] | PRR≥2, χ^2^≥4,  N≥3 |
| BCPNN | IC = log_2_^a(a + b + c + d)/[(a + c)(a + b)]^  IC025=e^ln(IC)-1.96(1/a+1/b+1/c+1/d)^0.5^ | IC025>0 |
| MGPS | EBGM = a(a + b + c + d)/(a + c)/(a + b)  EBGM05=e^ln(EBGM)-1.64(1/a+1/b+1/c+1/d)^0.5^ | EBGM05>2, N>0 |

Abbreviations: a: the number of reports with suspect adverse drug event (ADE) of the suspect drug; b: the number of reports with all other ADEs of the suspect drug; c: the number of reports with the suspect ADE of all other drugs; d: the number of reports with all other ADEs of all other drugs; ROR: reporting odds ratio; CI: confidence interval; N: the number of co-occurrences; PRR: proportional reporting ratio; χ^2^: chi-squared; BCPNN: Bayesian confidence propagation neural network; IC: information component; IC025: the lower limit of the 95% two-sided CI of the IC; MGPS: multi-item gamma Poisson shrinker; EBGM: empirical Bayesian geometric mean; EBGM05: the lower 95% one-sided CI of EBGM
